# Supplementary material for: Approach to map nanotopography of cell surface receptors
Source: Commun Biol. 2022 Mar 9;5:218. doi: 10.1038/s42003-022-03152-y (PMC8907216; doi:10.1038/s42003-022-03152-y)
Supplement: Supplementary file 2 — Supplementary Information [file 42003_2022_3152_MOESM2_ESM.pdf]

Supplementary Information

For the manuscript: Franke et al. Approach to map nanotopography of cell surface receptors

Contents:

Supplementary Figure S1. The impact of coverslip coating on cell surface morphology.

Supplementary Figure S2. Confocal laser scanning microscopy of CD4-GFP variants in Jurkat T cells.

Supplementary Figure S3. Glycine forms a narrow, gel-like structure on glass coverslips.

Supplementary Figure S4. Interference reflection microscopy (IRM) of T-cell contacts with coated coverslips.

Supplementary Figure S5. The impact of coverslip coating on surface morphology of Raji, RAW264.7 and COS-7 cells.

Supplementary Figure S6. The impact of fixation on surface morphology of cells immobilized on glycine-coated coverslips.

Supplementary Figure S7. Calcium response measurements on coverslips functionalized to stimulate (OKT3 antibody) or immobilize T cells (PLL and glycine).

Supplementary Figure S8. Non-specific stimulation of cultured and primary T cells by a coated surface of coverslips.

Supplementary Figure S9. Localization precision.

Supplementary Figure S10. Resolution improvement of dTRABI compared to TRABI determined by Fourier Ring Correlation (FRC).

Supplementary Figure S11. Segmentation of cells.

Supplementary Figure S12. Two-dimensional SMLM of tested T cell receptors immobilized on glycine-coated coverslips.

Supplementary Figure S13. Quantitative analysis of axial receptor distribution.

Supplementary Figure S14. Normalized localization count plots averaged over all ROIs for all cells per type.

Supplementary Figure S15. Exemplary selection of dTRABI images for CD4 WT, CD4 CS1 and CD45.

Supplementary Figure S16. Cell surface receptor nanotopography on a dying cell with extensive three-dimensional deformations visualized by three-dimensional dTRABI imaging.

Supplementary Figure S17. T-cell surface morphology on glycine-coated coverslips.

Supplementary Figure S18. The employed super-resolution method provides adequate information about native distribution of receptors on Jurkat cells.

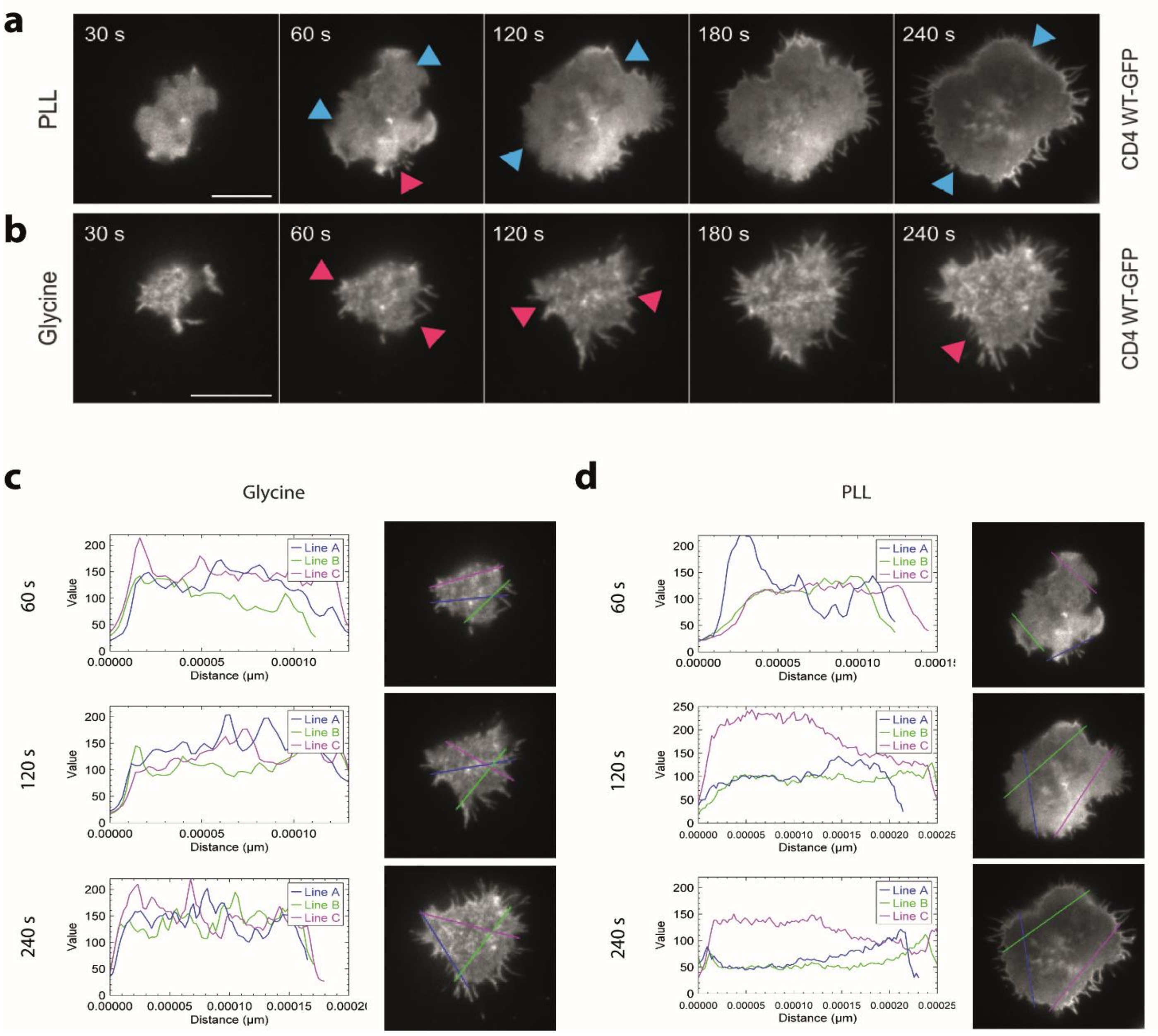

Supplementary Figure S1. The impact of coverslip coating on cell surface morphology. a-b) Live-cell TIRF microscopy of CD4-GFP in T cells landing on PLL-coated (a) or glycine-coated coverslips (b) measured at 37°C (see **Methods** and Supplementary Movies 1 and 2). Blue arrowheads indicate areas of rapid flattening of the cell surface and random distribution of CD4, as indicated by line-profiles in c-d. Red arrowheads indicate areas with heterogeneous distribution of CD4 on the cell surface as indicated by line-profiles (see below). Selected time points for the representative cells are shown. In total, 14 cells on glycine-coated and 10 cells on PLL-coated coverslips were analyzed. **c-d)** Two-dimensional images of T cell-coverslip contact sections (as in a and b) with indicated line selections (right panels) and the corresponding intensity line profiles (left panels). Snapshots acquired 60, 120 and 240 s after the first contact of the cell with the coverslip were analyzed.

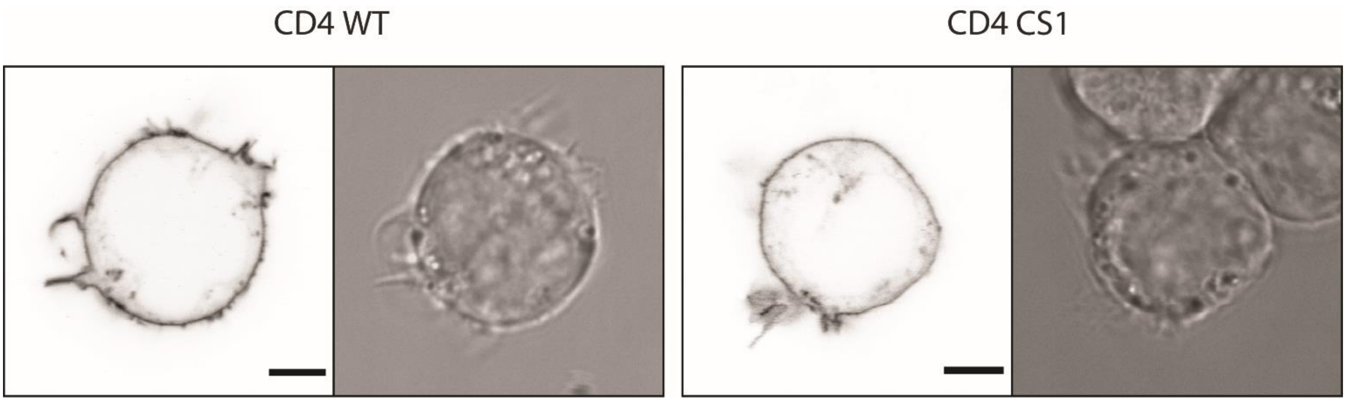

Supplementary Figure S2. Confocal laser scanning microscopy of CD4-GFP variants in Jurkat T cells. Live-cell confocal image of GFP fluorescence (midplane section; left panels) and brightfield (right panels) in Jurkat T cells transfected with CD4 WT GFP (left hand side) or CD4 CS1 GFP (right hand side) plasmids. Representative images are presented. In total, 46 (CD4 WT) and 41 cells (CD4 CS1) were imaged in five independent experiments.

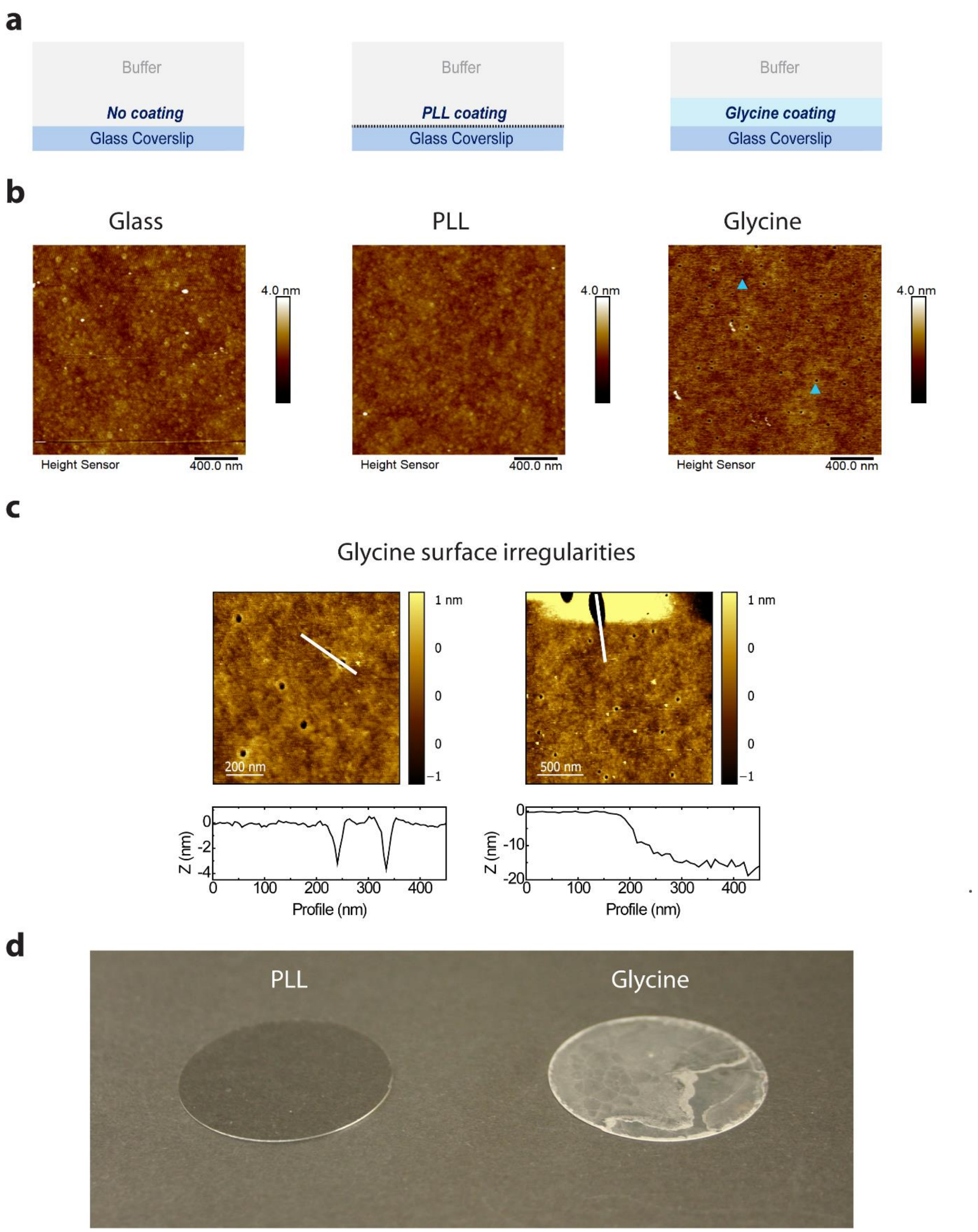

Supplementary Figure S3. Glycine forms a narrow, gel-like structure on glass coverslips. a) Schematic illustration of the coverslip coatings tested using atomic force microscopy (AFM). **b)** Comparison of the AFM topography images of the coverslip surface without further coating (left panel) and after coating with PLL (middle panel) or glycine (right panel). Blue arrowheads in the right-hand panel indicate two examples of small topography features (holes), which populate the surface of glycine-coated coverslips. **c)** Magnified AFM topography images on a glycine-coated coverslip (upper panels) with respective height profiles (lower panels) taken in the area with the smaller, almost perfectly circular holes (diameter ~30 nm, depth ~3–4 nm on the average; left panels), and in the area with a larger hole with the diameter >200 nm and depth >20 nm (right panels). **d)** Glycine coating of glass coverslips (right) forms a white precipitate after a brief drying, indicating a hydrogel formation. No such precipitate can be observed on the PLL-coated coverslips (left), which do not differ from the uncoated coverslips (not shown).

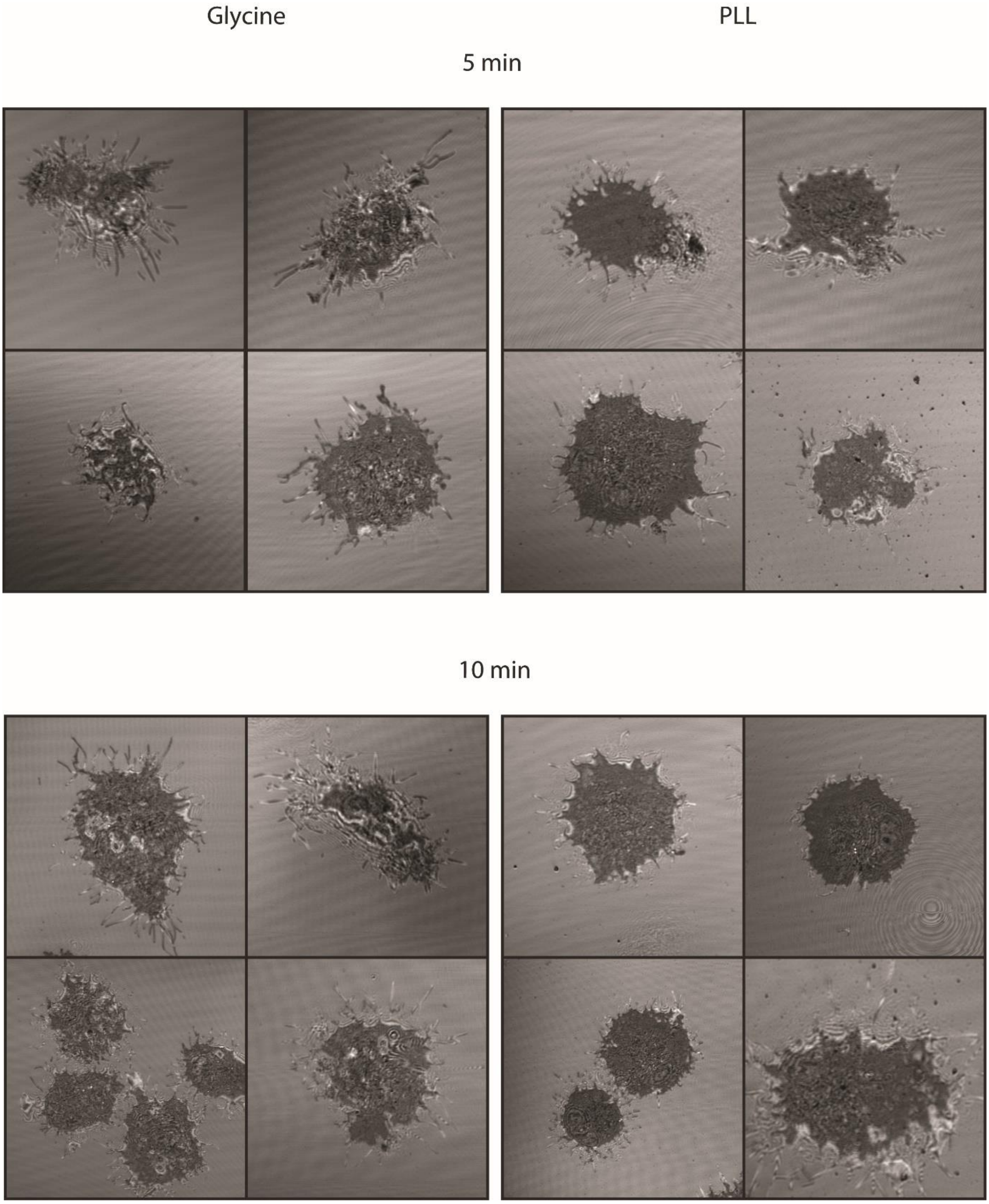

Supplementary Figure S4. Interference reflection microscopy (IRM) of T-cell contacts with coated coverslips. Live-cell IRM images of transfected Jurkat CD4 KO cells on glycine- (left hand side) or PLL-coated (right hand side) coverslips. Images were acquired 5 min or 10 min after injection of cells into the imaging chamber (see **Methods**). 16-25 cells were analyzed in three independent experiments. Four representative cells are presented.

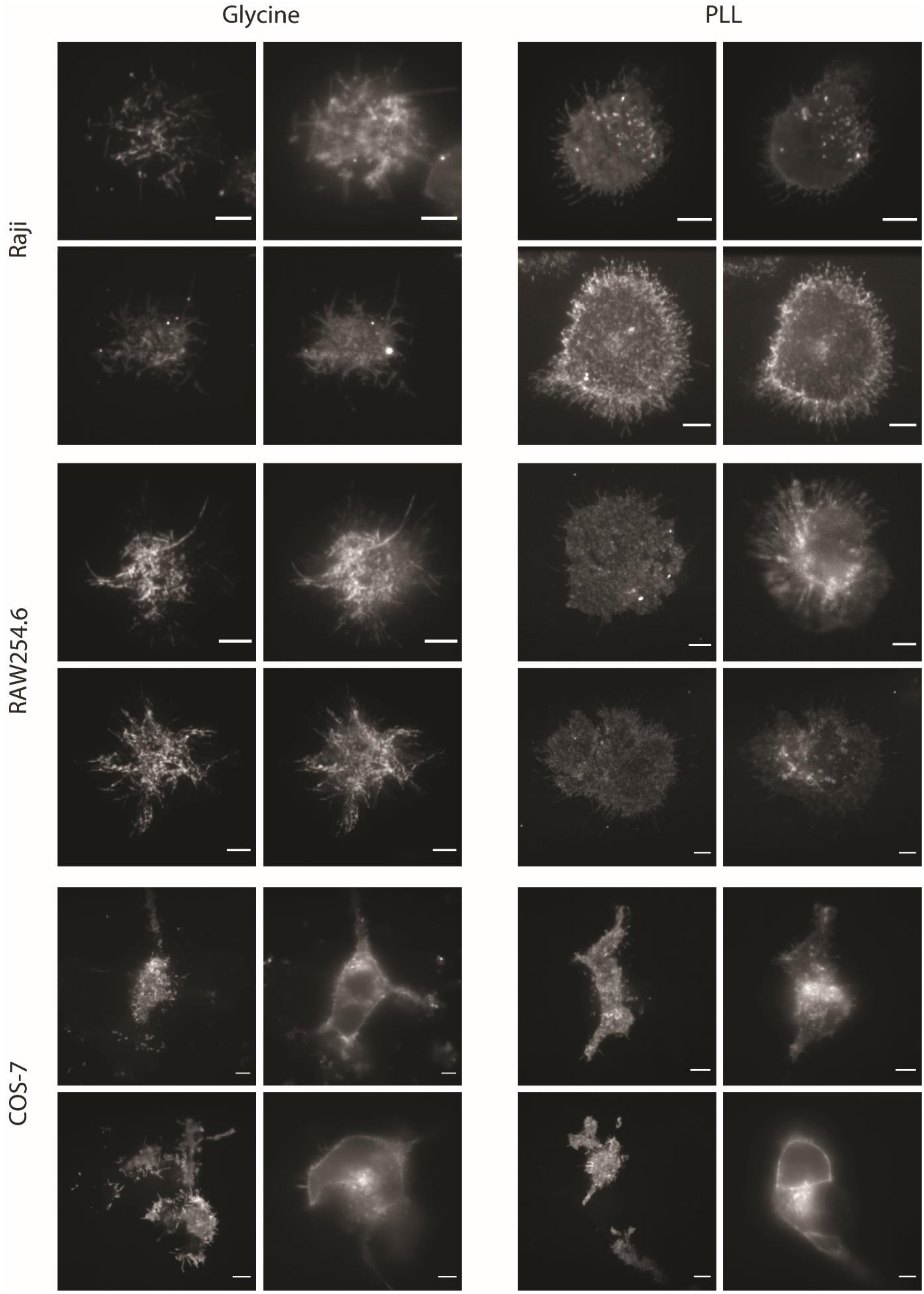

Supplementary Figure S5. The impact of coverslip coating on surface morphology of Raji, RAW264.7 and COS-7 cells. Cells transfected with CD4-GFP were immobilized on glycine- (left hand side) or PLL-coated (right hand side) coverslips and imaged using HILO illumination mode (see **Methods**). The cell contact with the coated optical surface is shown in the left panel, the cell body in the right panel. Two representative fluorescence images of cells are presented. 17 Raji cells, 17 RAW 264.7 cells and 14 COS-7 cells were analyzed in two independent experiments.

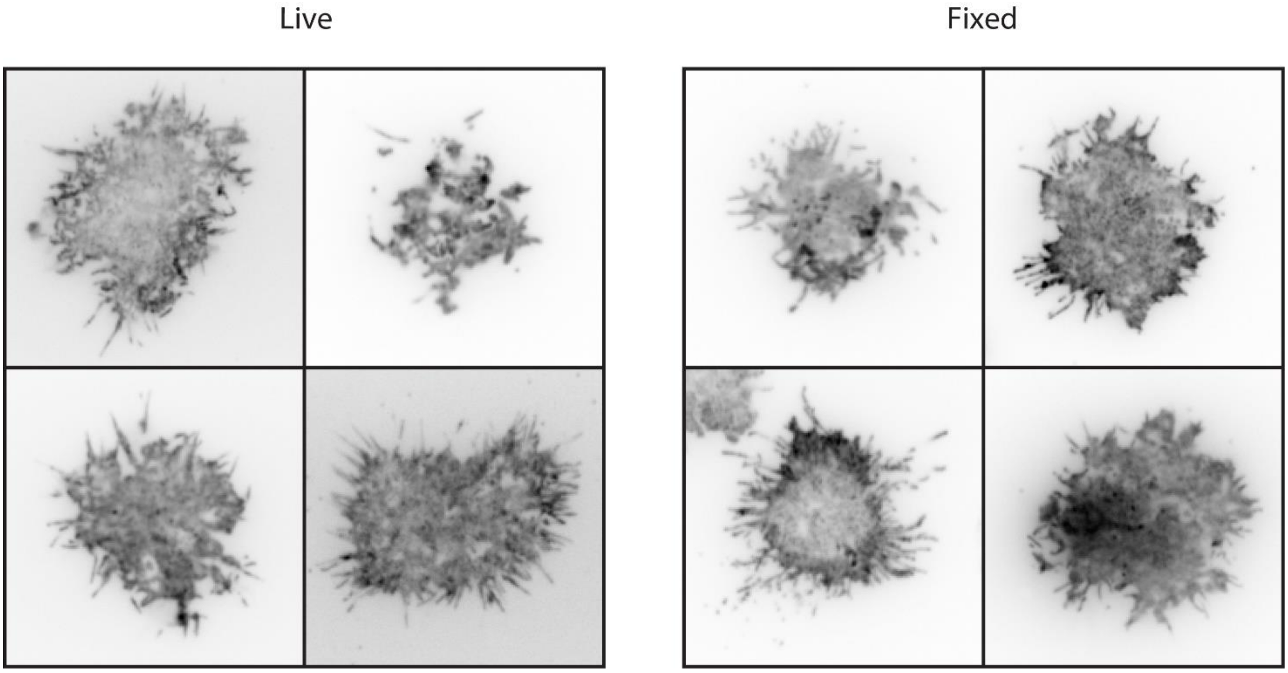

Supplementary Figure S6. The impact of fixation on surface morphology of cells immobilized on glycine-coated coverslips. Living (left hand side) and fixed (right hand side) Jurkat T cells transfected with CD4-GFP were imaged using HILO illumination mode (see **Methods**). The fluorescence of CD4-GFP, which is distributed evenly at the surface of T cells imaged with diffraction limited techniques, is presented. Four representative cells are presented. Over 50 cells were analyzed in three independent experiments.

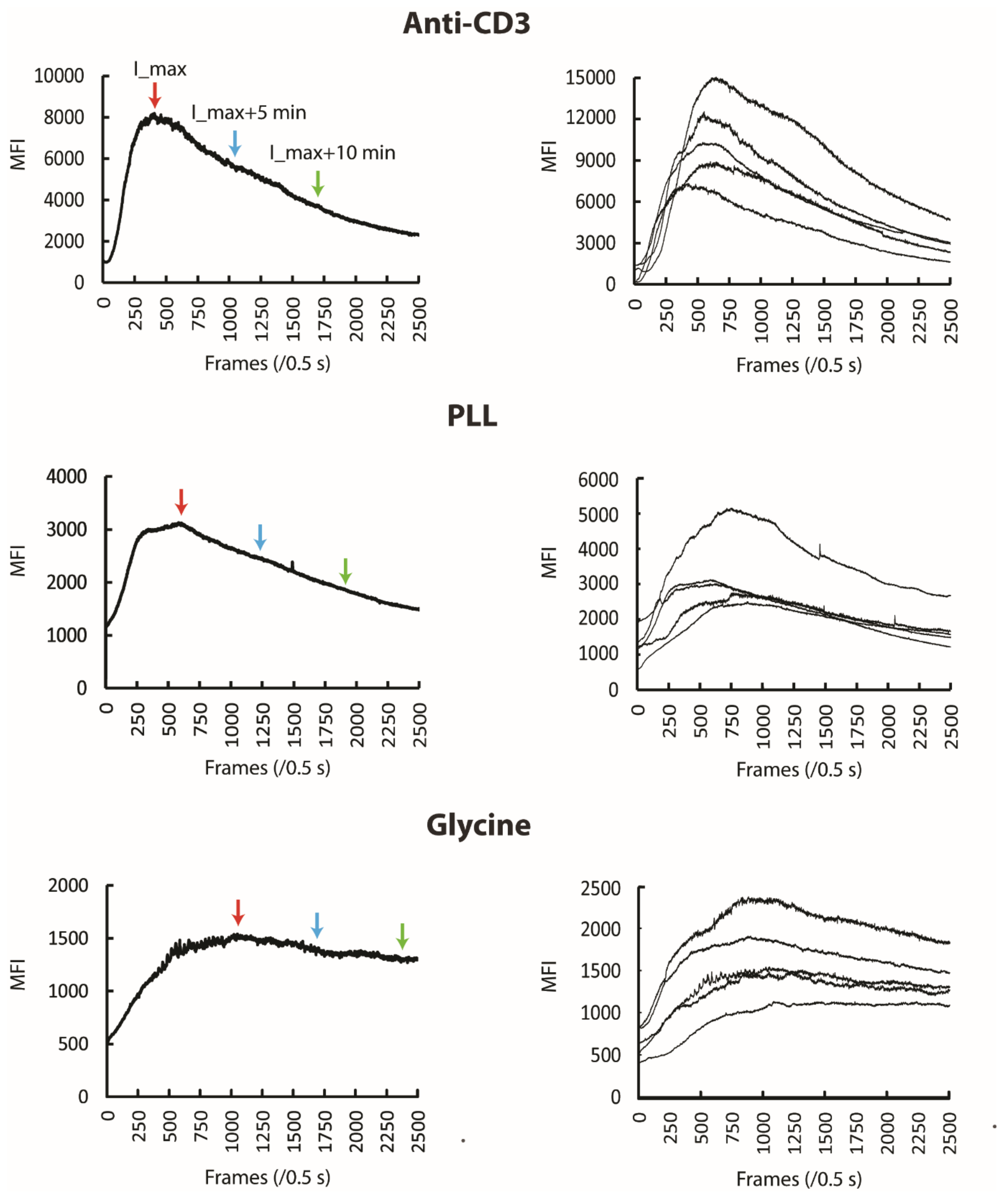

Supplementary Figure S7. Calcium response measurements on coverslips functionalized to stimulate (OKT3 antibody) or immobilize T cells (PLL and glycine). Representative time profiles of mean fluorescence intensity measured in T cells transfected with calcium sensor ($GCaMP6f_u$) using TIRF microscopy (2 fps) and illumination with 488 nm laser line are shown in the left panels. 11 cells were imaged on OKT3-antibody (upper panel), 16 cells on PLL- (middle panel) and 12 cells on glycine-coated (lower panel) coverslips. Cell footprints were manually selected at the time of maximum signal detection and the mean fluorescence of the area was determined for > 6 min using standard functions of Fiji/ImageJ software (version 1.52p; ref.[1]). Red arrows indicate maximum mean intensity (I_{max}), blue the signal detected 5 minutes later ($I_{max+5\ min}$) and green the signal detected after another 5 minutes ($I_{max+10\ min}$). The calcium response of cells interacting with the functionalized surface was calculated as $I_{max}/I_{max+5\ min}$ and $I_{max}/I_{max+10\ min}$ as summarized in Fig.1f. The $I_{max}/I_{max+5\ min}$ and $I_{max}/I_{max+10\ min}$ values thus indicate transientness of the signal in tested cells. However, the use of fast responding, fast decaying, plasma membrane-associated sensor allowed to monitor the cellular calcium response without the access to the on-rate data. Five tracks representing the maximal signal diversity are presented on the right-hand side.

a

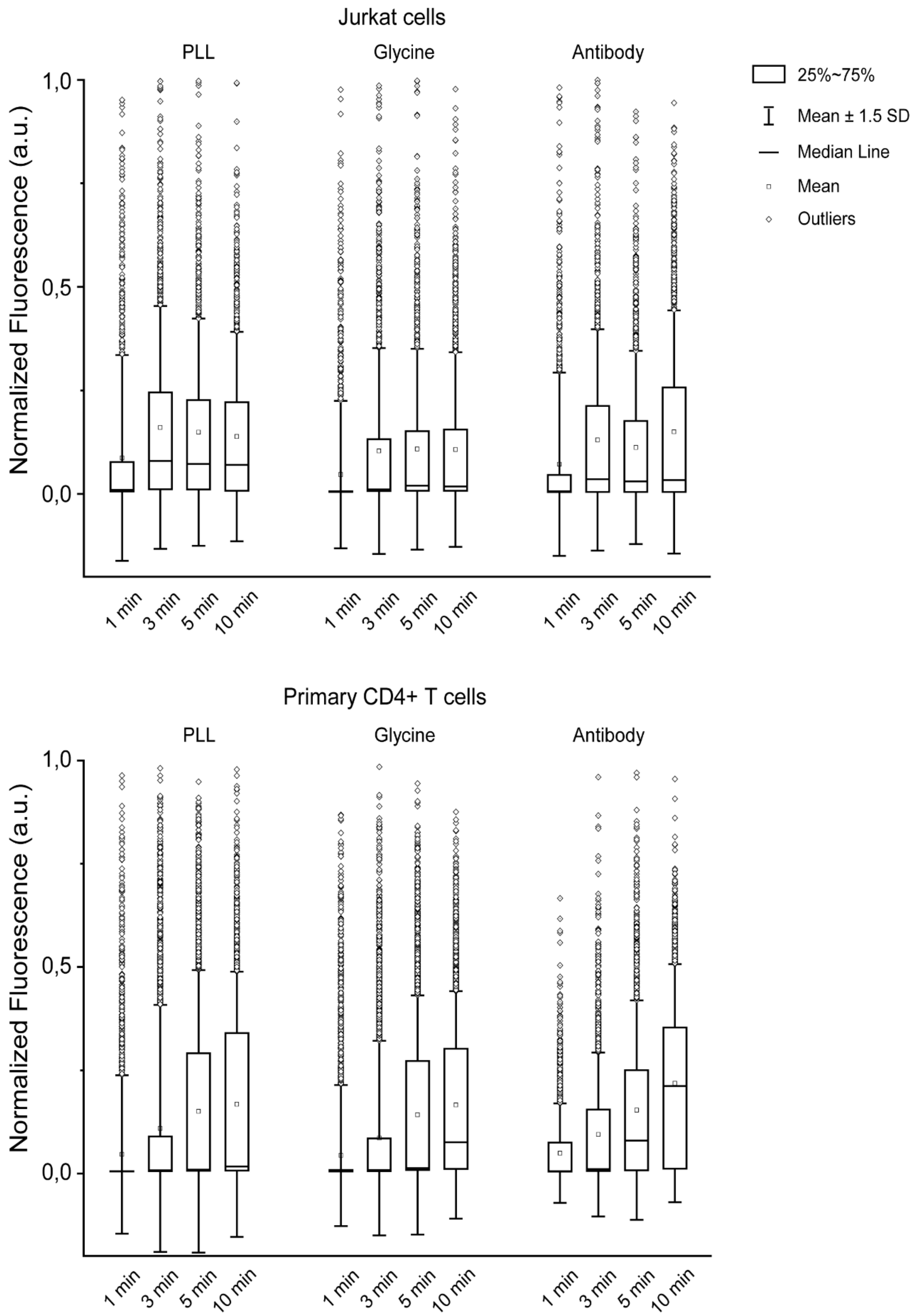

b

Supplementary Figure S8. (continues on the next page)

c

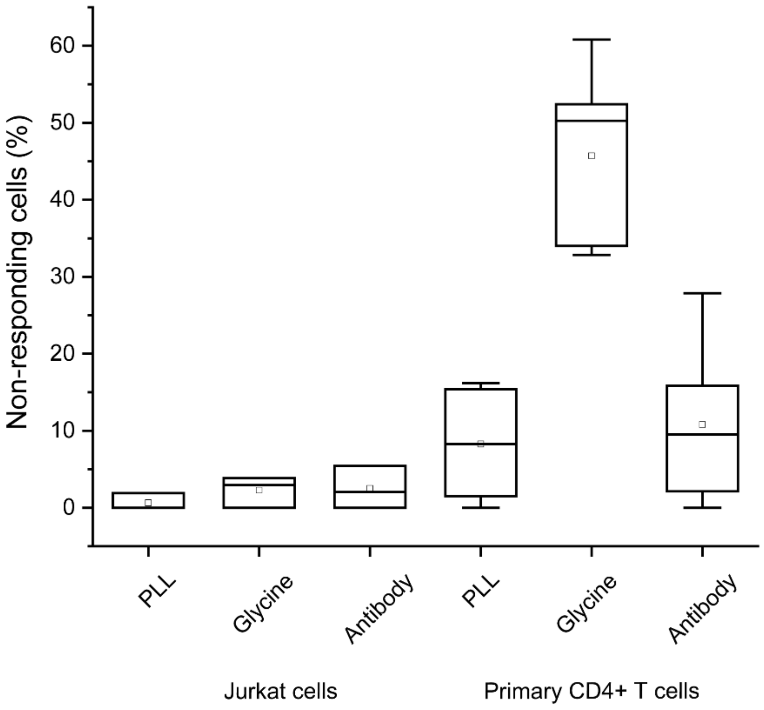

Supplementary Figure S8. Non-specific stimulation of cultured and primary T cells by a coated surface of coverslips. a) Calcium mobilization in Jurkat cells was monitored during their immobilization on PLL-, glycine- and antibody-coated coverslips. Fluorescence intensity of calcium-sensitive dye Fluo-4 was acquired for 1691 (PLL), 2040 (glycine) and 1856 cells (C305; anti-TCR antibody). The graph presents normalized (relative) intensity with respect to the maximal response induced by 1 µM ionomycin at the end of each measurement. **b**) Calcium mobilization in primary human CD4+ T cells was monitored during their immobilization on PLL-, glycine- and antibody-coated coverslips. Fluorescence intensity of calcium-sensitive dye Fluo-4 was acquired for 3249 (PLL), 5162 (glycine) and 2626 cells (C305; anti-TCR antibody). The graph presents normalized (relative) intensity with respect to the maximal response induced by 1 µM ionomycin at the end of each measurement. **c)** Box-plot graph presenting a fraction of cells, which did not mobilize calcium during the whole period of the measurement (15 min) after their landing on PLL, glycine and antibody-coated coverslips. Both, Jurkat and primary CD4+ T cells were evaluated. Data were processed using CalQuo2 algorithm [2] and represent 3-7 independent experiments.

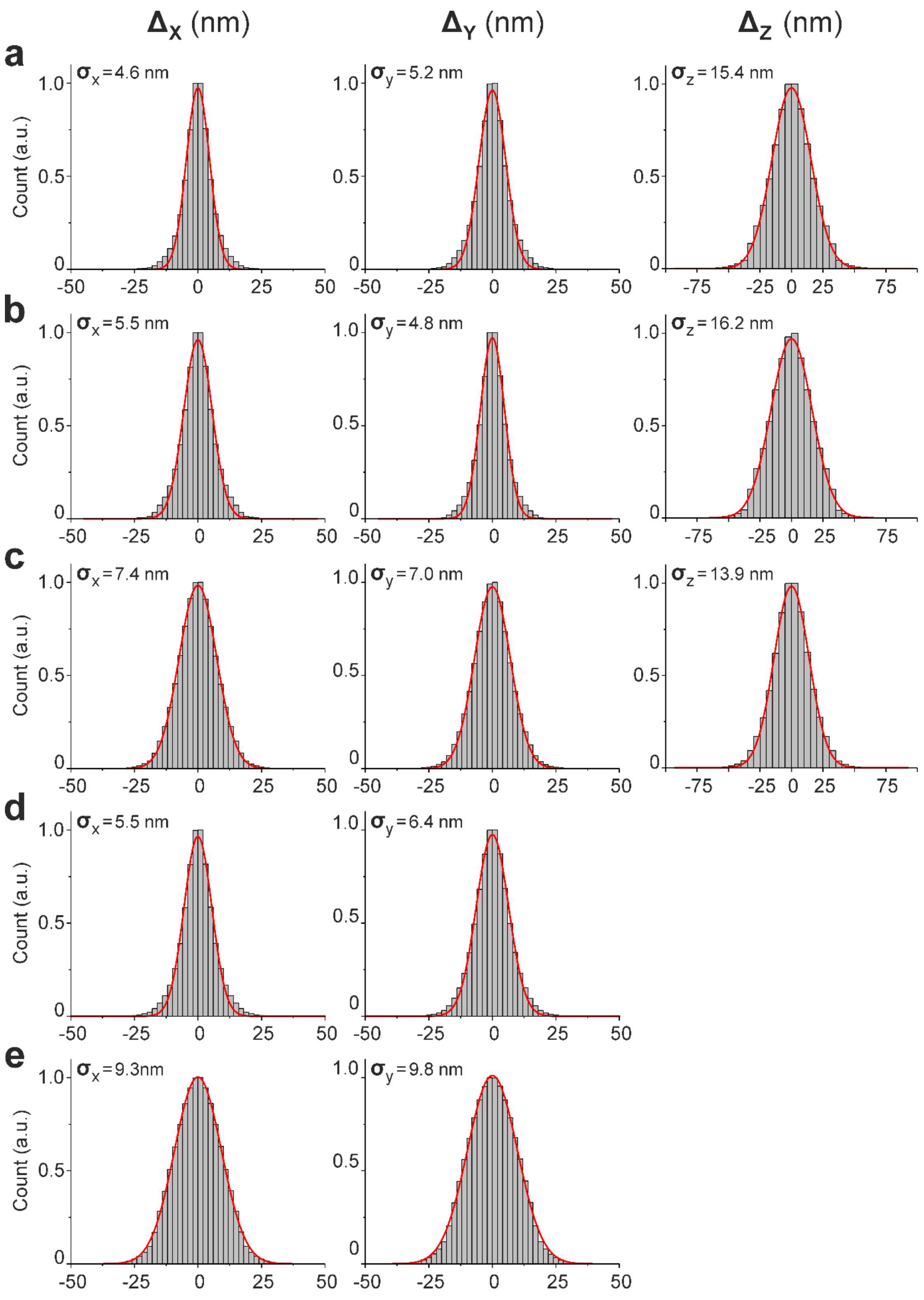

Supplementary Figure S9. Localization precision. Normalized histograms of spatio-temporal nearest neighbor tracks, constituting the three-dimensional localization precision of the dTRABI and two-color SMLM measurements (see **Methods**). x-, y- and z- were individually determined and are stated as the standard deviation of the respective distribution as numerical value **σ**. Sample and imaging modalities: **a)** dTRABI of CD4 WT (Alexa Fluor 647) **b)** dTRABI of CD4 CS1 (Alexa Fluor 647) **c)** dTRABI of CD45 (Alexa Fluor 647) **d)** two-dimensional *d*STORM of CD45 (Alexa Fluor 647) **e)** two-dimensional PALM of CD45 (mEos2).

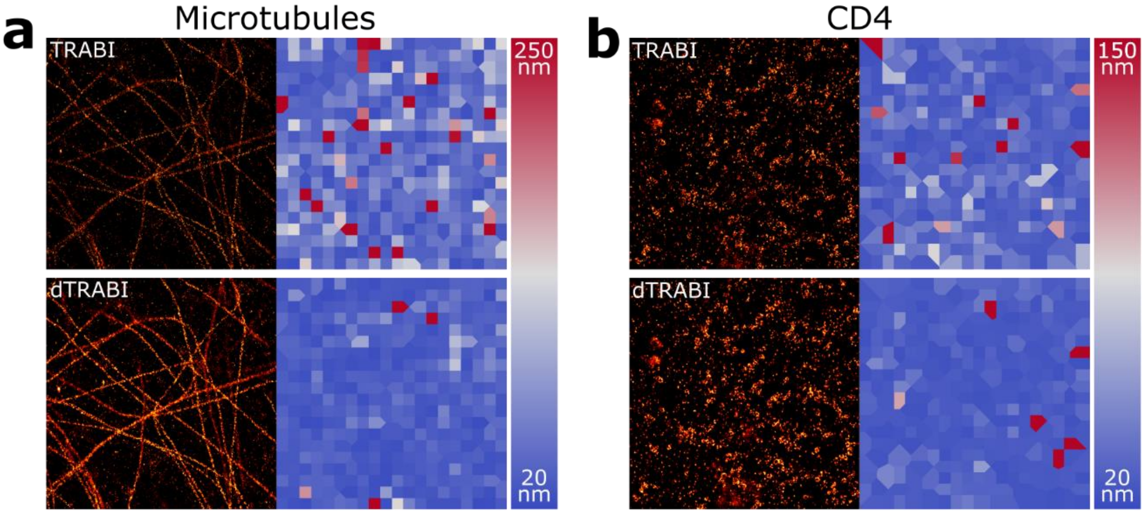

c

FRC resolution (nm)	Microtubules TRABI	Microtubules dTRABI	CD4 TRABI	CD4 dTRABI
**Median**	**49.1**	**33.7**	**30.9**	**26.9**
**MAD**	15.0	5.5	5.3	2.2
**Mean**	78.6	40.1	42.4	32.4
**SD**	117.5	35.5	50.0	30.6
**Max**	1139.5	430.3	490.3	340.9
**Min**	21.4	21.3	22.9	22.9

Supplementary Figure S10. Resolution improvement of dTRABI compared to TRABI determined by Fourier Ring Correlation (FRC). a) Representative three-dimensional dSTORM images of microtubules (data from ref.[3]). The three-dimensional localization sets were derived according to either the standard TRABI-Biplane algorithm (TRABI, *top*) or the new dTRABI approach (*bottom*). The according 2D-FRC maps, visualizing the local FRC-resolution, are depicted on the right. We selected the median 2D (i.e., x-y) FRC resolution of the images as the most robust resolution metric, which was derived to 49 nm (TRABI) and 34 nm (dTRABI). Overall, dTRABI improved the structural resolution by 30 percent. **b**) Representative three-dimensional dSTORM images of CD4 (data reanalyzed from **Fig. 5a**). The three-dimensional localization sets were derived according to either the standard TRABI-Biplane algorithm (TRABI, *top*) or the dTRABI approach (*bottom*). The according FRC maps are depicted on the right. The median 2D FRC resolution of the images was derived to 31 nm (TRABI) and 27 nm (dTRABI). dTRABI hence improved the structural resolution by 13 percent. FRC analysis was performed with NanoJ-Squirrel[4], additional relevant FRC metrics are listed in **c**). Scale bars, 1 µm.

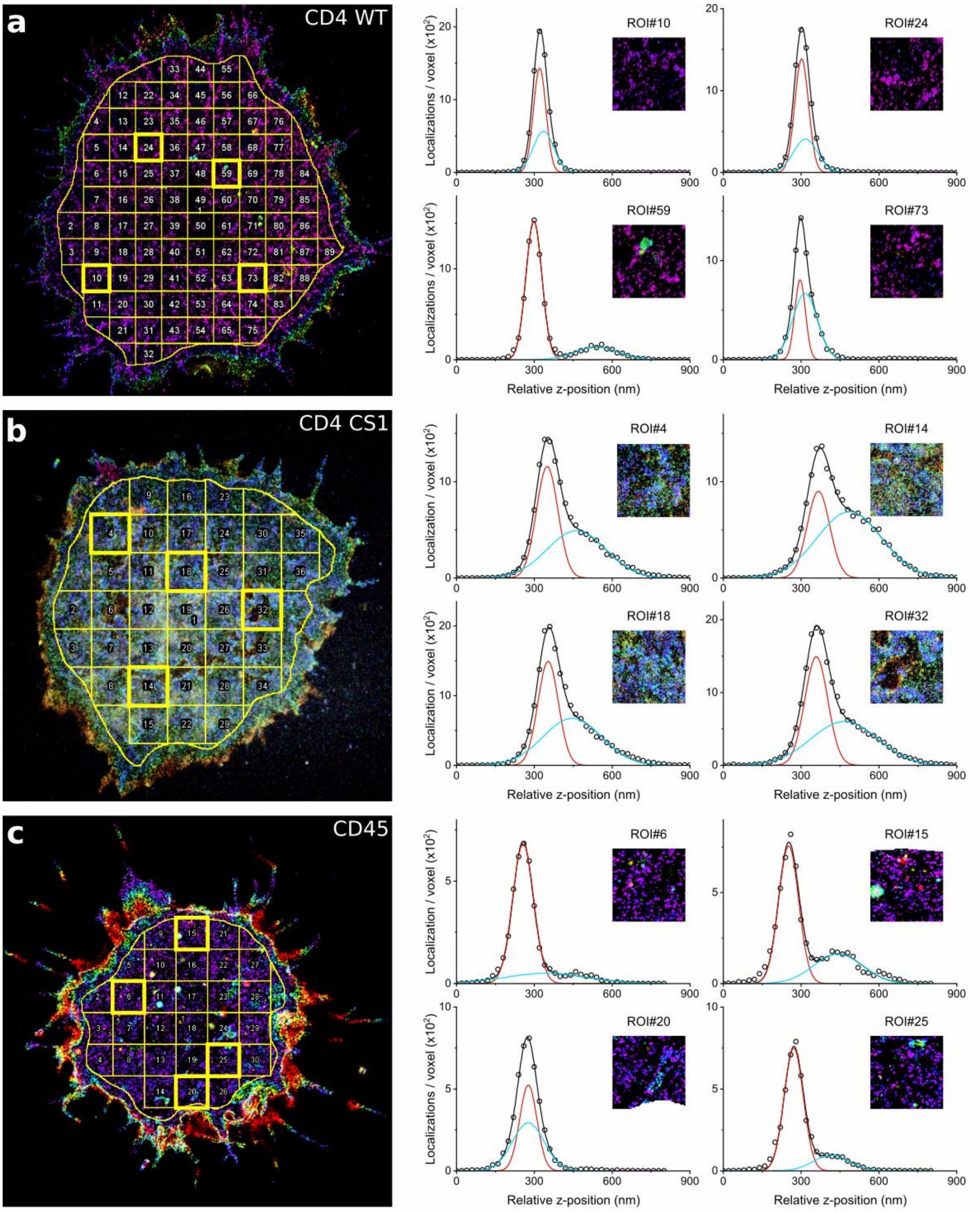

Supplementary Figure S11. Segmentation of cells. For quantitative axial analysis of surface receptors, the interior of each cell footprint was first manually selected (ROI#1) to avoid the impact of cell edges. Afterwards, the area was further segmented into squared 2 µm × 2 µm ROIs. In border areas, ROIs were kept if the area was ≥75% of 4 µm² (see **Methods**). **a**) Segmentation and quantitative axial analysis for CD4 WT. *Left*: The cell as shown in Fig. 5a segmented, *right*: example ROIs and Gaussian fitting for quantitative analysis of receptor z-distribution as in Fig.5e-g. **b**) Segmentation and quantitative axial analysis for CD4 CS1. *Left*: The cell as shown in Fig. 5c segmented, *right*: example ROIs and Gaussian fitting for quantitative analysis of receptor z-distribution. **c**) Segmentation and quantitative axial analysis for CD45. *Left*: The cell as shown in Fig. 4b segmented, *right*: example ROIs and Gaussian fitting for quantitative analysis of receptor z-distribution. Selected ROIs are depicted with a bold frame in the whole-cell images on the left-hand side. In graphs with the axial receptor distribution, black circles represent raw data, black line the bi-Gaussian fit, which is the sum of two Gaussians as depicted in red and blue.

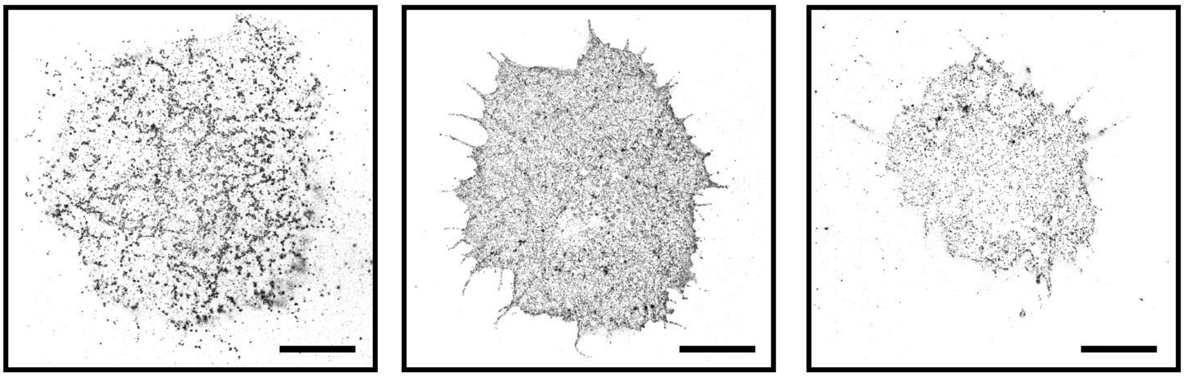

Supplementary Figure S12. **Two-dimensional SMLM of tested receptors immobilized on glycine-coated coverslips.** dSTORM images of CD4 WT (left), CD4 CS1 (middle) and CD45 (right) on the surface of unstimulated T cells. The two-dimensional images indicate different nanoscopic organization of the receptors but cannot be analyzed quantitatively due to a three-dimensional character of the T-cell surface. Both, native CD4 (CD4 WT) and its non-palmitoylatable mutant (CD4 CS1) are expressed mainly on the cell surface (see Supplementary Fig. S2). Scale bars, 5 µm.

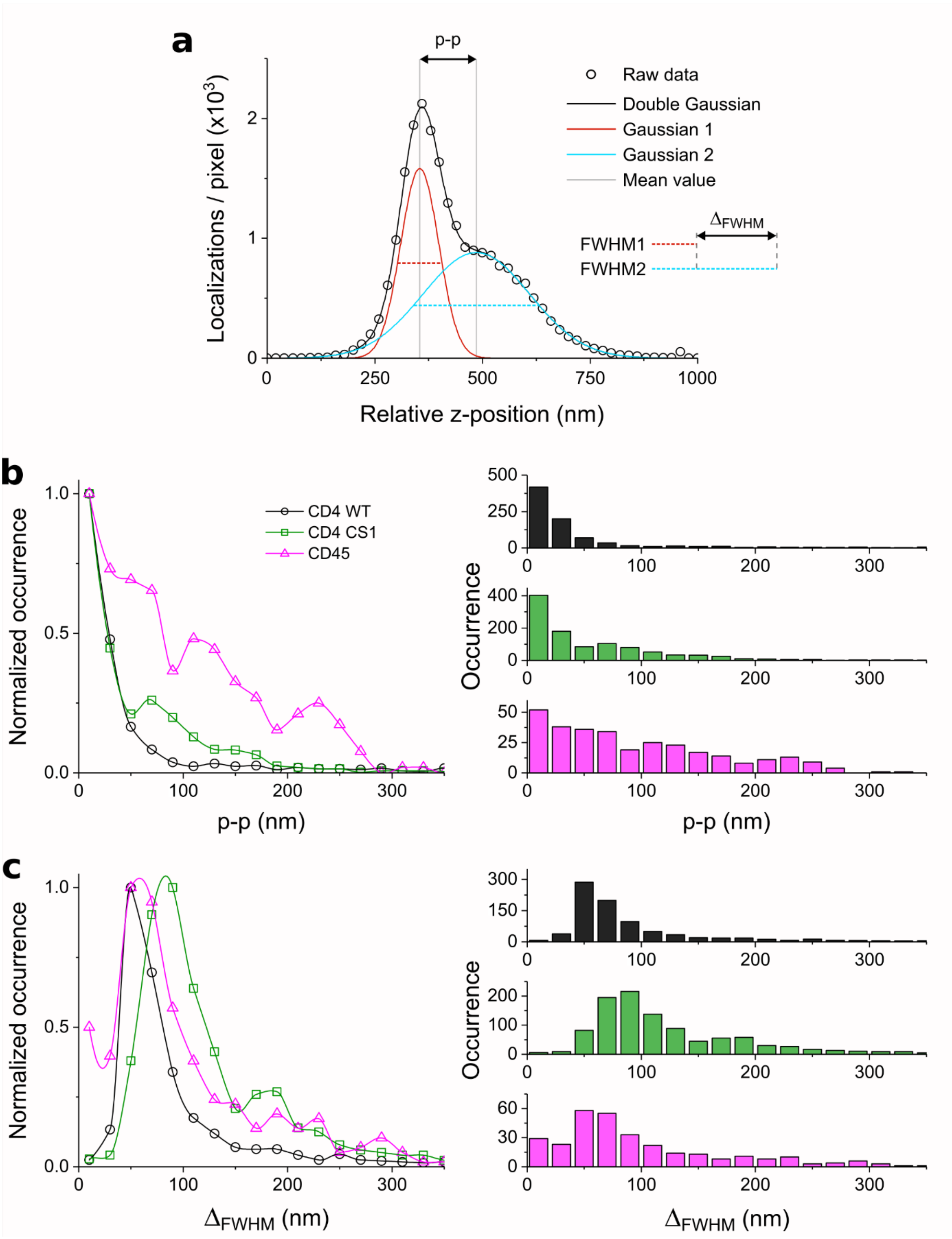

Supplementary Figure S13. Quantitative analysis of axial receptor distribution. a) The principle of data analysis using a bi-Gaussian fit to derive the quantitative parameters peak-to-peak distance (p-p) and width difference ($_{FWHM}$). **b**) p-p, which represents the absolute value of the difference the mean values as indicated in a) (p-p = mean2-mean1). **c**) $_{FWHM}$, which represents the absolute value of the difference between the FWHM values of the two Gaussians as indicated in a) ($_{FWHM}$ = FWHM2-FWHM1). The normalized distributions are plotted on the left and histograms with absolute values on the right-hand side. In b and c, black represents data for CD4 WT, green for CD4 CS and magenta for CD45. All data points were spline interpolated to guide the eye. For CD4 WT 21 cells, CD4 CS1 18 cells and CD45 13 cells were analyzed.

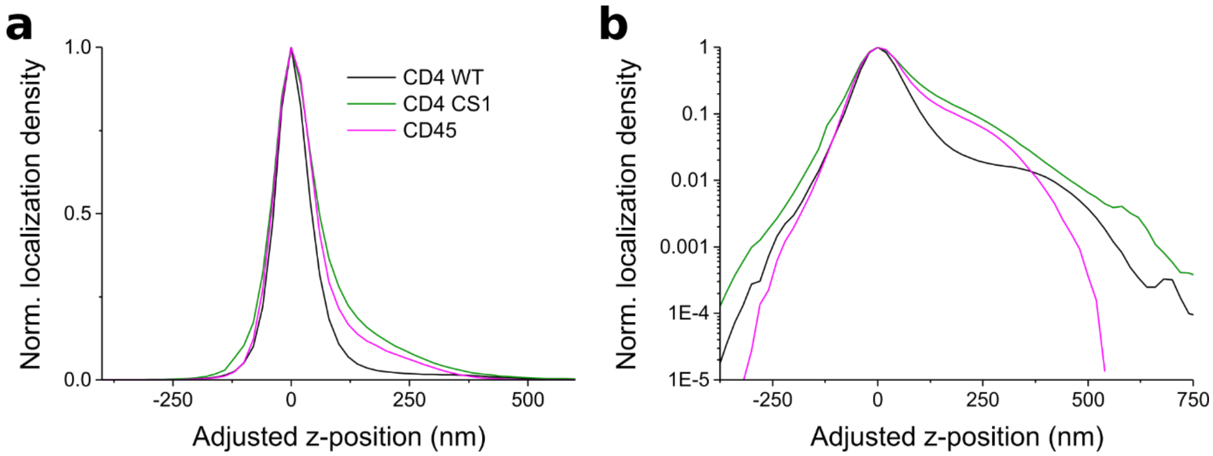

Supplementary Figure S14. Normalized localization count plots averaged over all ROIs for all cells per type. a) All localizations of the manual selection of each cell (ROI#1 in each image, cf. Supplementary Fig. S11) were plotted as distribution along the z-axis, which was adjusted according to the mean value of the first Gaussian (Gaussian 1 in Fig. 5e). The aligned distributions of all ROIs per cell were subsequently summed up, leading to the global distribution for each tested receptor. **b**) Logarithmic plot of a). In total, 7.48×10^6, 8.76×10^6 and 1.40×10^6 localizations were averaged for CD4 WT, CD4 CS1 and CD45, respectively.

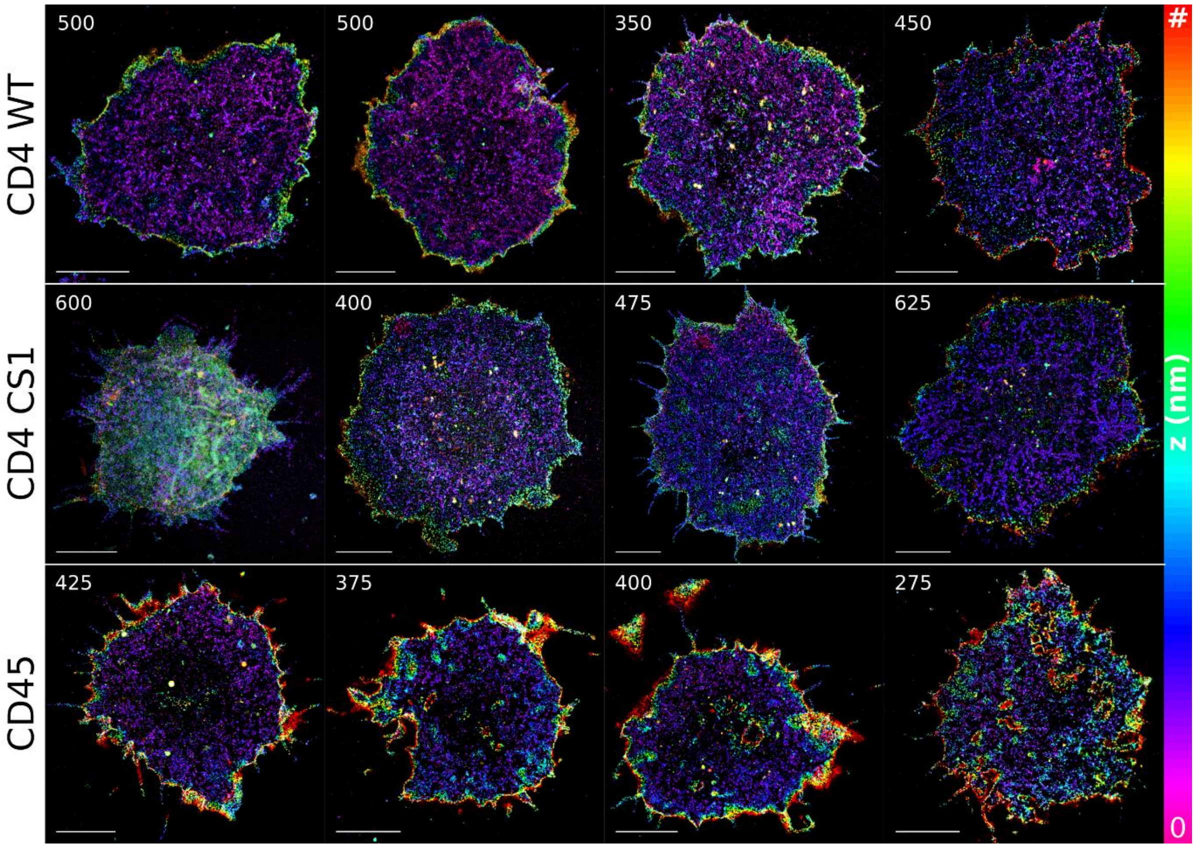

Supplementary Figure S15. Exemplary selection of dTRABI images for CD4 WT, CD4 CS1 and CD45. The maximum z-value is depicted for each cell (top left corner), e.g., for the cell in the upper right corner the z-range is from 0 to 450 nm. Marker '#' in the color-bar means the maximum z-value for each cell. In total, 21, 18 and 13 cells were analyzed for CD4 WT, CD4 CS1 and CD45, respectively. Scale bars, 5 µm.

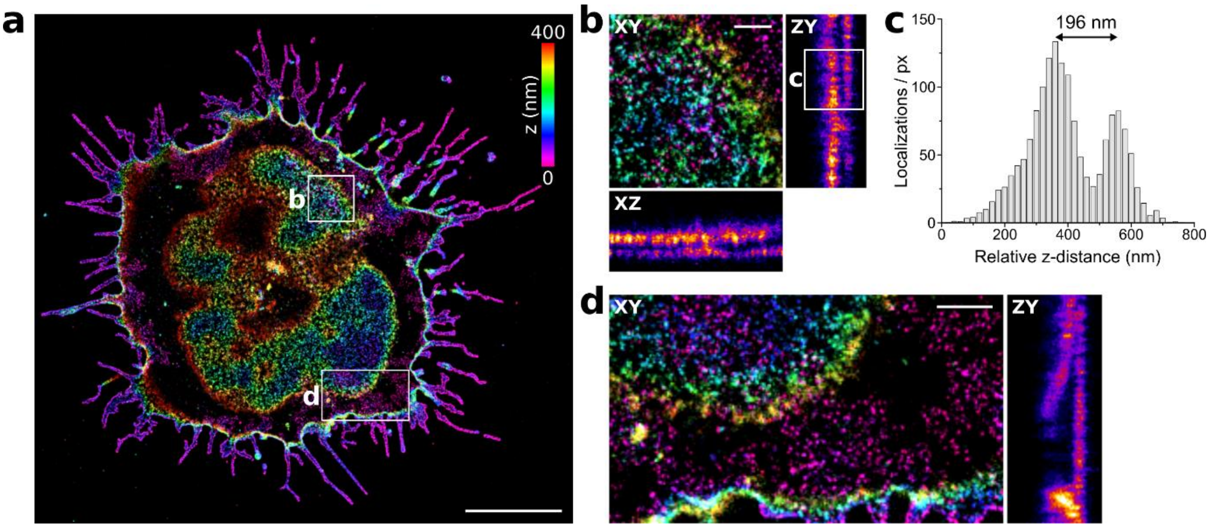

Supplementary Figure S16. Cell surface receptor nanotopography on a dying cell with extensive three-dimensional deformations visualized by three-dimensional dTRABI imaging. a) Three-dimensional dTRABI image of CD4 with selected ROIs exhibiting broad z-distribution of receptor localizations. **b)** Magnified x-y, x-z and z-y projections of the ROI indicated in a). **c)** The axial distribution of localizations as depicted in the y-z plot in b). **d)** Magnified x-y and z-y projections of the ROI indicated in a). Scale bars, a) 5 µm (x, y), b, d) 500 nm (x, y, z).

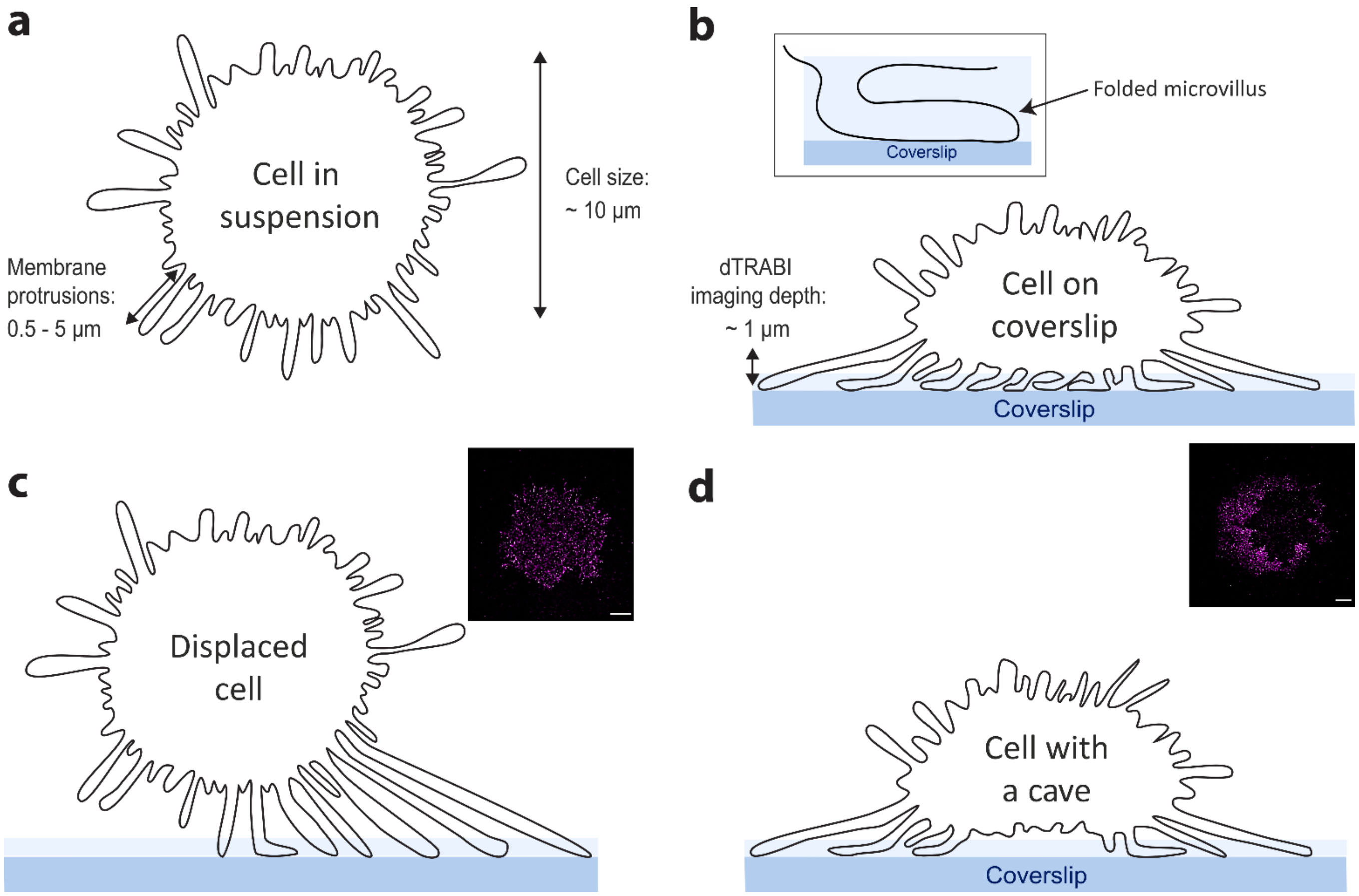

Supplementary Figure S17. T-cell surface morphology on glycine-coated coverslips. a) Schematic illustration of a T cell in suspension. The cell is covered with numerous membrane protrusions of different sizes. The size of Jurkat T cells is, on average, 10 µm in diameter, the protrusions, even though a majority are small (0.5–2 µm length), can extend up to 5 µm from the cell body. **b)** After landing on a glycine-coated coverslip, membrane protrusions of T cells fold under the cell body but are not rapidly removed as on PLL. **c-d)** With the axial penetration depth of dTRABI being ~1 µm, we were unable to detect receptors at the plasma membrane basis of T cells exhibiting displacement of the cell body from the contact site (c) or those with the cavity formed between the cell body and the coated coverslip. The footprints of such cells show discontinuous labeling or a hole in the middle of the image (see the inserts). The blue stripes represent glass coverslip, the light blue stripes above, the glycine layer (not to scale).

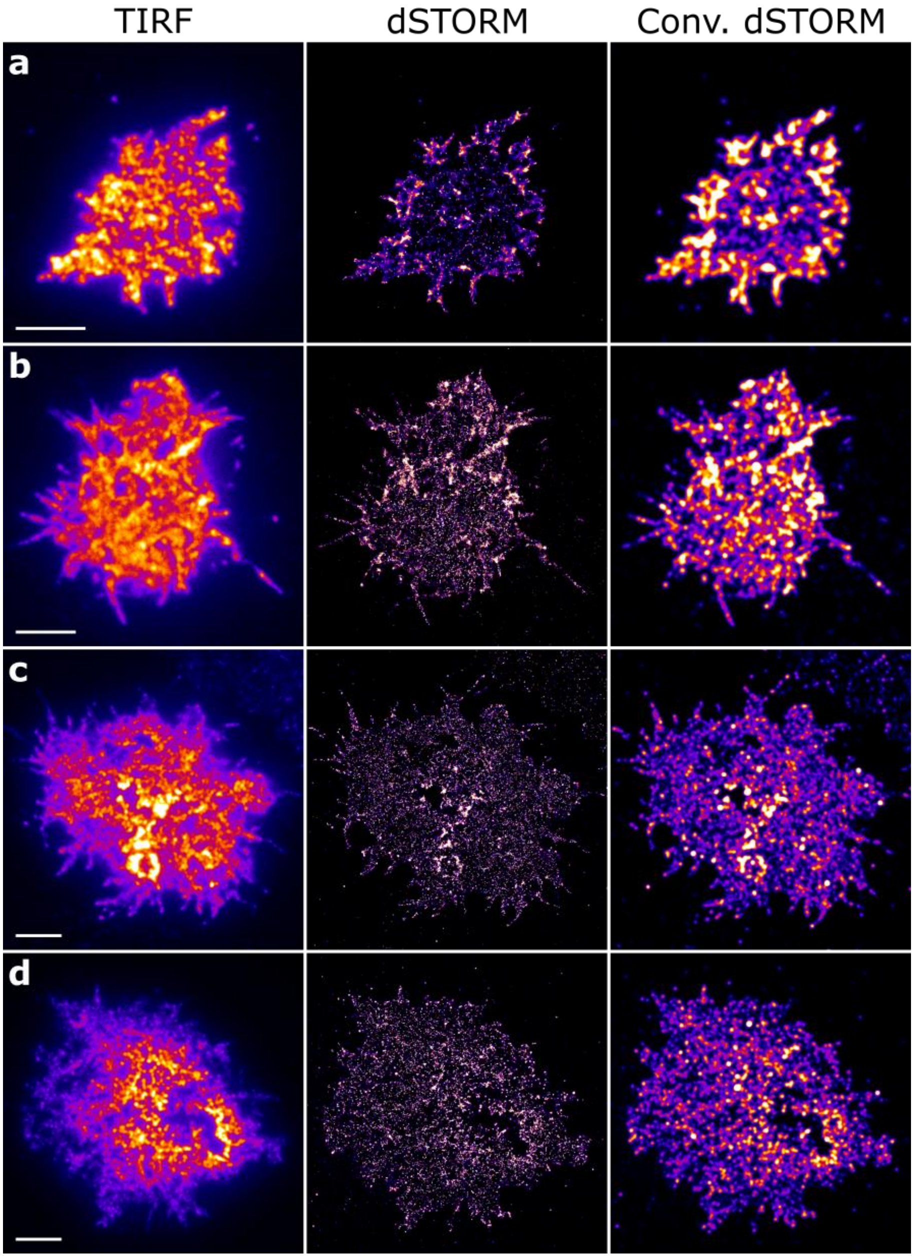

Supplementary Figure S18. **The employed super-resolution method provides adequate information about native distribution of receptors on Jurkat cells.** *Left panel*: the conventional TIRF image acquired prior to the dSTORM acquisition, *middle*: the reconstructed dSTORM image, and *right*: the Gaussian-convolved dSTORM image. dSTORM images were convolved with a 2D Gaussian function with the standard deviation set to 132 nm. **a)-d** Four representative Jurkat cells labelled with anti-CD4 antibody (OKT4) and imaged using our SMLM setup (see **Methods**) are presented. Scale bars corresponds to 5 µm.

Supplementary References

1. Schindelin, J. *et al.* Fiji: an open-source platform for biological-image analysis. *Nat Methods* **9**, 676-682 (2012).

2. Lee, A.M., Colin-York, H. & Fritzsche, M. CalQuo (2) : Automated Fourier-space, population-level quantification of global intracellular calcium responses. *Sci Rep* **7**, 5416 (2017).

3. Franke, C., Sauer, M. & van de Linde, S. Photometry unlocks 3D information from 2D localization microscopy data. *Nat Methods* **14**, 41-44 (2017).

4. Culley, S. *et al.* Quantitative mapping and minimization of super-resolution optical imaging artifacts. *Nat Methods* **15**, 263-266 (2018).
