# Supplementary material for: Approach to map nanotopography of cell surface receptors
Source: Commun Biol. 2022 Mar 9;5:218. doi: 10.1038/s42003-022-03152-y (PMC8907216; doi:10.1038/s42003-022-03152-y)
Supplement: Supplementary file 3 — Description of Additional Supplementary Files [file 42003_2022_3152_MOESM3_ESM.pdf]

Description of Additional Supplementary Files

File name: Supplementary Movie 1
Description: Live cell TIRF microscopy of CD4-GFP in Jurkat cells landing on PLL-coated coverslips measured at 37°C. Representative cell as in Fig. 1c shown. Acquisition rate: 1 fps; video fame rate: 15 fps; scale bar: 5 µm. The image sequence was corrected for the photobleaching; the brightness and contrast were leveled using plugins of Fiji software.

File name: Supplementary Movie 2
Description: Live cell TIRF microscopy of CD4-GFP in Jurkat cells landing on glycinecoated coverslips measured at 37°C. Representative cell as in Fig. 1d shown. Acquisition rate: 1 fps; video fame rate: 15 fps; scale bar: 5 µm. The image sequence was corrected for the photobleaching; the brightness and contrast were leveled using plugins of ImageJ/Fiji software.

File name: Supplementary Movie 3
Description: Live cell microscopy of the Jurkat cells mobility on glycine-coated coverslips measured at 37°C. Phase contrast imaging using 20x objective was used. Acquisition rate: 20 fps; video fame rate: 75 fps; total time: 15 min. The brightness and contrast were leveled using plugins of ImageJ/Fiji software. Three independent experiments were measured.

File name: Supplementary Movie 4
Description: Live cell microscopy of the Jurkat cells mobility on PLL-coated coverslips measured at 37°C. Phase contrast imaging using 20x objective was used. Acquisition rate: 20 fps; video fame rate: 75 fps; total time: 15 min. The brightness and contrast were leveled using plugins of ImageJ/Fiji software. Three independent experiments were measured.

File name: Supplementary Movie 5
Description: Live cell microscopy of the Jurkat cells mobility on serum-coated coverslips measured at 37°C. Coverslips were coated with serum-containing medium prior to imaging (see Methods). Phase contrast imaging using 20x objective was used. Acquisition rate: 20 fps; video fame rate: 75 fps; total time: 15 min. The brightness and contrast were leveled using plugins of ImageJ/Fiji software. Three independent experiments were measured.

File name: Supplementary Data 1
Description: Source data generated and/or analyzed during this study.
