# Supplementary material for: Approach to map nanotopography of cell surface receptors
Source: Commun Biol. 2022 Mar 9;5:218. doi: 10.1038/s42003-022-03152-y (PMC8907216; doi:10.1038/s42003-022-03152-y)
Supplement: Supplementary file 10 — Reporting Summary [file 42003_2022_3152_MOESM10_ESM.pdf]

nature research

Corresponding author(s): Christian Franke, Sebastian van de Linde, Marek Cebecauer

Last updated by author(s): Jan 18, 2022

Reporting Summary

Nature Research wishes to improve the reproducibility of the work that we publish. This form provides structure for consistency and transparency in reporting. For further information on Nature Research policies, see our Editorial Policies and the Editorial Policy Checklist.

Statistics

For all statistical analyses, confirm that the following items are present in the figure legend, table legend, main text, or Methods section.

n/a	Confirmed	
☐	☒	The exact sample size (*n*) for each experimental group/condition, given as a discrete number and unit of measurement
☐	☒	A statement on whether measurements were taken from distinct samples or whether the same sample was measured repeatedly
☒	☐	The statistical test(s) used AND whether they are one- or two-sided<br>*Only common tests should be described solely by name; describe more complex techniques in the Methods section.*
☒	☐	A description of all covariates tested
☒	☐	A description of any assumptions or corrections, such as tests of normality and adjustment for multiple comparisons
☐	☒	A full description of the statistical parameters including central tendency (e.g. means) or other basic estimates (e.g. regression coefficient) AND variation (e.g. standard deviation) or associated estimates of uncertainty (e.g. confidence intervals)
☒	☐	For null hypothesis testing, the test statistic (e.g. *F*, *t*, *r*) with confidence intervals, effect sizes, degrees of freedom and *P* value noted<br>*Give P values as exact values whenever suitable.*
☒	☐	For Bayesian analysis, information on the choice of priors and Markov chain Monte Carlo settings
☒	☐	For hierarchical and complex designs, identification of the appropriate level for tests and full reporting of outcomes
☒	☐	Estimates of effect sizes (e.g. Cohen's *d*, Pearson's *r*), indicating how they were calculated

Our web collection on statistics for biologists contains articles on many of the points above.

Software and code

Policy information about availability of computer code

Data collection	commercial: AndorSolis, NIS Elements version 4.51
Data analysis	commercial: Origin2017; localization analysis: rapidSTORm 3.2 (methods); ImageJ/Fiji macros TRABI 1.2 , uManager, LocFileVisualizer for axial quantification (both open source download from http://bcp.phys.strath.ac.uk/photophysics/super-resolution/software/), biplane workflow programmed in python based on the published scripts (https://doi.org/10.1038/nmeth.4073), SOFI analyses as published in https://doi.org/10.1038/s41467-017-01857-x; and CalQuo2 software from authors of 10.1038/s41598-017-05322-z<br>the authors will provide software support upon personal request as practiced previously e.g. for all software related to https://doi.org/10.1038/nmeth.4073

For manuscripts utilizing custom algorithms or software that are central to the research but not yet described in published literature, software must be made available to editors and reviewers. We strongly encourage code deposition in a community repository (e.g. GitHub). See the Nature Research guidelines for submitting code & software for further information.

Data

Policy information about availability of data

All manuscripts must include a data availability statement. This statement should provide the following information, where applicable:

- Accession codes, unique identifiers, or web links for publicly available datasets
- A list of figures that have associated raw data
- A description of any restrictions on data availability

All raw data is available from the authors upon request.

Field-specific reporting

Please select the one below that is the best fit for your research. If you are not sure, read the appropriate sections before making your selection.

☒ Life sciences ☐ Behavioural & social sciences ☐ Ecological, evolutionary & environmental sciences

For a reference copy of the document with all sections, see nature.com/documents/nr-reporting-summary-flat.pdf

Life sciences study design

All studies must disclose on these points even when the disclosure is negative.

Sample size	For quantitative analyses at least 10 independent fields of views/cells were collected to account for heterogeneity. Evaluated cell numbers are stated in the text and in Methods.
Data exclusions	No non-inherent (to the experimental setup) data exclusion was performed. Within the SMLM workflow there exist a number of standard threshold and band-pass criteria which are all specified (methods); cells were selected based on their expression level and shape descriptors to exclude apoptotic cells (predetermined).
Replication	All results were consistently replicated in independent experiments. The cell numbers and replications are declared in the appropriate text of the manuscript. Samples can be prepared for the independent evaluation.
Randomization	Randomization was not relevant to this study.
Blinding	Blinding was not relevant to this study.

Reporting for specific materials, systems and methods

We require information from authors about some types of materials, experimental systems and methods used in many studies. Here, indicate whether each material, system or method listed is relevant to your study. If you are not sure if a list item applies to your research, read the appropriate section before selecting a response.

Materials & experimental systems

n/a	Involved in the study	
☐	☒	Antibodies
☐	☒	Eukaryotic cell lines
☒	☐	Palaeontology and archaeology
☒	☐	Animals and other organisms
☒	☐	Human research participants
☒	☐	Clinical data
☒	☐	Dual use research of concern

Methods

n/a	Involved in the study	
☒	☐	ChIP-seq
☒	☐	Flow cytometry
☒	☐	MRI-based neuroimaging

Antibodies

Antibodies used	Anti-CD45 (human; MEM28; source: ExBio), anti-CD4 (human; OKT4; source: Biolegend)
Validation	All batches of the directly labeled antibodies (with Alexa Fluor 647) were tested on CD4+CD45+ T cells and control cells using flow cytometry.

Eukaryotic cell lines

Policy information about cell lines

Cell line source(s)	Jurkat (E6 clone), RAW264.7, Raji and COS-7 from ATCC and CD4 knock-out made in the laboratory (see Methods).
Authentication	the main surface markers. CD4 negativity of the knock-out line was re-evaluated every 12 weeks.
Mycoplasma contamination	The cell lines used in this study were regularly tested for the mycoplasma contamination were negative in all cases.
Commonly misidentified lines (See ICLAC register)	n.a.
